# Elevated CO_2_ reduced antimony toxicity in wheat plants by improving photosynthesis, soil microbial content, minerals, and redox status

**DOI:** 10.3389/fpls.2023.1244019

**Published:** 2023-09-13

**Authors:** Galal Khamis, Ahmed Mohamed Reyad, Emad A. Alsherif, Mahmoud M. Y. Madany, Shereen Magdy Korany, Han Asard, Hamada AbdElgawad

**Affiliations:** ^1^ Department of Laser Applications in Metrology, Photochemistry, and Agriculture (LAMPA), National Institute of Laser Enhanced Sciences, Cairo University, Giza, Egypt; ^2^ Botany and Microbiology Department, Faculty of Science, Beni-Suef University, Beni-Suef, Egypt; ^3^ Biology Department, College of Science, Taibah University, Al-Madinah Al-Munawarah, Saudi Arabia; ^4^ Department of Biology, College of Science, Princess Nourah bint Abdulrahman University, Riyadh, Saudi Arabia; ^5^ Integrated Molecular Plant Physiology Research, Department of Biology, University of Antwerp, Antwerp, Belgium

**Keywords:** antimony, eCO2, wheat, antioxidants, photosynthesis, minerals contents

## Abstract

**Introduction:**

Antimony (Sb), a common rare heavy metal, is naturally present in soils at low concentrations. However, it is increasingly used in industrial applications, which in turn, leads to an increased release into the environment, exerting a detrimental impact on plant growth. Thus, it is important to study Sb effects on plants under the current and future CO_2_ (eCO_2_).

**Methods:**

To this end, high Sb concentrations (1500 mg/kg soil) effects under ambient (420 ppm) and eCO_2_ (710 ppm) on wheat growth, physiology (photosynthesis reactions) and biochemistry (minerals contents, redox state), were studied and soil microbial were evaluated.

**Results and discussion:**

Our results showed that Sb uptake significantly decreased wheat growth by 42%. This reduction could be explained by the inhibition in photosynthesis rate, Rubisco activity, and photosynthetic pigments (Cha and Chb), by 35%, 44%, and 51%, respectively. Sb significantly reduced total bacterial and fungal count and increased phenolic and organic acids levels in the soil to decrease Sb uptake. Moreover, it induced oxidative markers, as indicated by the increased levels of H_2_O_2_ and MDA (1.96 and 2.8-fold compared to the control condition, respectively). To reduce this damage, antioxidant capacity (TAC), CAT, POX, and SOD enzymes activity were increased by 1.61, 2.2, 2.87, and 1.86-fold, respectively. In contrast, eCO_2_ mitigated growth inhibition in Sb-treated wheat. eCO_2_ and Sb coapplication mitigated the Sb harmful effect on growth by reducing Sb uptake and improving photosynthesis and Rubisco enzyme activity by 0.58, 1.57, and 1.4-fold compared to the corresponding Sb treatments, respectively. To reduce Sb uptake and improve mineral availability for plants, a high accumulation of phenolics level and organic acids in the soil was observed. eCO_2_ reduces Sb-induced oxidative damage by improving redox status. In conclusion, our study has provided valuable insights into the physiological and biochemical bases underlie the Sb-stress mitigating of eCO_2_ conditions. Furthermore, this is important step to define strategies to prevent its adverse effects of Sb on plants in the future.

## 1 Introduction

Rare heavy metals are released into the environment due to human activities such as urbanization, industrialization, land-use change, rock deterioration, mining, and the use of agrochemicals. High accumulations of these heavy metals reduce plant production and endanger human health through biomagnification in food chains (Mohammed et al., 2011). Antimony (Sb) is one of the rare heavy metals that negatively affect plant growth, and it is not considered a vital nutrient. It is naturally found in two oxidation states and is absorbed by plants from the soil. Sb occurs naturally in soils, sedimentary rocks, and water with concentrations of 0.3-8.6 mg/kg, 0.15-2 mg/kg, and 1 µg/mL, respectively (Filella et al., 2001). It has biochemical properties similar to arsenic and bismuth, as they are in the same group in the periodic table, classified as metalloid and exhibit multiple oxidation states (Filella et al., 2001; Babula et al., 2008). Sb pollution has caused considerable alarm in many parts of the world. According to the United States Environmental Protection Agency, and the European Union, antimony and its compounds are designated as priority pollutants and are toxic to humans and rats (Liang et al., 2018). Human exposure to Sb is controlled, with a maximum of 5 µg/L in drinking water and 0.04 mg Sb/kg for plastic materials, while Sb is toxic to humans at chronic absorption rates of more than 100 mg/day, while rats are susceptible to dosages ranging from 11 to 75 mg/day (Filella et al., 2001). Although, Sb is naturally present in soils at low concentrations. It is increasingly used in industrial applications (Nishad and Bhaskarapillai, 2021), which in turn, leads to an increased release into the environment, exerting a detrimental impact on plant growth.

Heavy metal absorption, transport, and sequestration are all important aspects of plant life when it comes to coping with heavy metal toxicity. Since heavy metals’ physicochemical properties are similar to those of essential microelements, plasma membrane transporters are present in roots to aid in their absorption (DalCorso et al., 2013). In ecosystem, Sb cycling through the food web is an essential part of the ecosystem’s nutrient flow, it can serve as a trace element required for the growth and metabolism of certain microorganisms and it is recently considered an emerging environmental pollutant (Bolan et al., 2022). Given the importance of Sb in the ecosystem, research into its phytotoxicity and alleviation in plants is critical, as plants are primary producers and may transport Sb to higher trophic levels. However, there has been little research on the processes of antimony uptake, transport, and toxic impact (Miravet et al., 2005) and biological toxicity and bioavailability (Liang et al., 2018). For instance, Sb exposure has been proven to inhibit rice and wheat germination (Shtangeeva et al., 2012). A recent study found that high concentrations of Sb at 250 mg/kg soil reduced *Acorus calamus* growth (Zhou et al., 2018). A decrease in photosynthesis is the likely cause of the observed growth inhibition due to Sb. Zhou et al. (2018) found that when plants were exposed to high concentrations of Sb stress, their net photosynthetic rate (Pn), leaf pigments, and PSII electron transfer quantum yield rate all dropped by a lot. Also, Luo et al. (2021) found that high levels of Sb in plant cells can interact with the sulfhydryl group of chloroplast proteins, which can change the way chloroplasts are built and how they work. As a consequence, this interference resulted in decreased photosynthetic efficiency, ultimately leading to a decline in plant growth. Overall, Sb is a common contaminant in the environment; however, there are few studies on Sb phytotoxicity on plant growth, physiology, and metabolism (Duan et al., 2023).

Heavy metals at high concentrations impair electron transport chain performance, as well as mitochondrial and chloroplast function, and disturb redox equilibrium, eventually leading to the generation of reactive oxygen species (ROS) (Cuypers et al., 2016). By using both enzymatic and non-enzymatic antioxidant defenses (Sofo et al., 2004; Khamis et al., 2019; AbdElgawad et al., 2020), plants can make defenses against oxidative stress caused by free radicals.Plants employ various strategies such as producing protective metal-binding metabolites, storing metal chelates in vacuoles or releasing them into the rhizosphere (AbdElgawad et al., 2020). Plants trigger multiple forms of phytochemicals in plant tissues, in addition to the antioxidant’s arsenal defense, by allocating their primary less active carbohydrate to structural carbohydrates and amino acids to bioactive secondary metabolites when these conditions occur (Almuhayawi et al., 2020). Thus, to enhance plant stress tolerance against Sb toxicity, reduced Sb uptake and improved redox status are crucial.

Crops will be exposed to high amounts of CO_2_ in the atmosphere as well as increasing soil metal toxicity in the future. Interestingly, eCO_2_ improves biomass accumulation, photosynthetic rates, and agricultural output when water and nutrients are abundant (Sharkey et al., 2007). The mitigation of the effect of eCO_2_ on heavy metals was also reported (Li et al., 2010). For instance, eCO_2_ alleviated As toxicity in maize and barley (AbdElgawad et al., 2021) and mitigated the arsenic stress in wheat and soybean. Positive plant responses to eCO_2_ can be explained by improved antioxidant capabilities and/or photosynthetic efficiency (AbdElgawad et al., 2022). In this regard, eCO_2_, on the other hand, mitigates the deleterious impacts of abiotic conditions like heavy metals, in part by up-regulating antioxidant defense metabolism and decreasing photorespiration, thus lowering oxidative stress (AbdElgawad et al., 2016). High CO_2_ increased the resales of root exudates such as organic acids (Xiong et al., 2019). This in turn induced a reduction in soil pH, which decreased the bioavailability of heavy metals, increasing the consequences of soil metal poisoning (Guo et al., 2012). Overall, to forecast the impact of future climate change on agricultural growth and production, crop responses to soil metal toxicity under increasing CO_2_ levels must be investigated.

Wheat (*Triticum* spp.) is one of the staple cereal grains thatis used in many nations around the world to feed both people and animals. It contributes significantly to human growth and diet by offering proteins, carbs, and some inorganic micronutrients. Worldwide, moderate regions with substantial agricultural areas are used to raise cereal grains. The world commerce in wheat is bigger than that for any other cereal crop because it ranks third overall in worldwide output (UN; Anita et al., 2010). Therefore, investigating the effects of Sb phytotoxicity on wheat plant growth under current and future climate is needed. To the best of our knowledge, no in-depth studies on the Sb stress mitigation impact of eCO_2_. So, our study explored the mechanisms that wheat plants use to cope with Sb toxicity. Investigating the interaction between Sb and high CO_2_ levels is essential for comprehending the complex dynamics of environmental pollution with Sb, climate change, and their consequences for sustainable agriculture. Understanding these interactions can aid in developing strategies to ensure food security and sustainable agriculture.

## 2 Material and methods

### 2.1 Experimental setup

For 20 minutes, a homogeneous lot of wheat was surface sterilized in 5% (v/v) sodium hypochlorite. In 20 *25 cm pots, a soil potting mix (Tref EGO substrates, Moerdijk, Netherlands) was placed. The pots were then put in a controlled growth room (16/8 h day/night photoperiod, light intensity of 150 mol m^-2^ s^-1^, 60/70% humidity, and 21/18°C air temperature). Wheat (*Triticum aestivum* L. cv Giza 157) plants were treated to the following conditions: 1) ambient CO_2_ (aCO_2_, 410 ppm); 2) elevated CO_2_ (eCO_2_, 720 ppm); 3) Sb (1500 mg/Kg soil); and 4) eCO_2_ + Sb (1500 mg/Kg soil). Wheat cultivar was selected because of its sensitivity to abiotic stress including heavy metals. The climate scenarios ‘current’ and ‘future climate’ were chosen according to the IPCC-SRES B2-scenario prediction of moderate change for the year 2100 (IPCC, 2012). Moreover, The intermediate scenario (RCP 4.5 scenario) considers of CO_2_ to 720 ppm (an intermediate scenario) (The Intergovernmental Panel on Climate Change, 2022). The Sb levels used were chosen after preliminary studies on the effect of varied Sb concentrations (100-1500 mg/Kg soil) on wheat growth. The control and treatment pots were watered daily until the soil water content reached 60% (SWC). Plants were taken six weeks following the Sb/CO_2_ treatment and divided into roots and shoots. The tissues were kept at -80 for subsequent biochemical analysis. Soils were also collected at the end of the experiment for chemical analysis.

### 2.2 Mineral content evaluation

About 200 mg of each wheat-treated shoot was digested in an oven in an HNO_3_/H_2_O solution (5:1 v/v). The inductively coupled plasma mass spectrometry (ICP-MS, Finnigan Element XR, and Scientific, Bremen, Germany) was used to quantify the amounts of macro minerals and trace elements at 25°C, with nitric acid in 1% employed as standards. (AbdElgawad et al., 2019).

### 2.3 Soil analysis

After gently shaking the roots, 10 g of rhizosphere soil was swirled in double distilled water. The total phenolic content of the soil extract was determined spectrophotometrically using Zhang et al. (2006) technique. Citric acid was determined by HPLC, as described by De Sousa et al. (2016). According to Zhang et al. (2006), ten grams of rhizosphere were forcefully mixed in distilled water, and the filtrates were used to spectrophotometrically assess the phenolic content (Shimadzu UV-Vis 1601 PC, Japan). To measure the negative effect of heavy metals on microbial population in the soil, ten grams of each soil sample was added to 90 mL of 0.1% (w/v) solution of sodium pyrophosphate. After homogenization for 30 min, this solution was decimally diluted (10^−1^ to 10^−7^) and aliquots of the resulting solutions were plated on appropriate culture media. After incubation at 25 or 30°C, for up to 10 days, the colony-forming units (CFUs) were counted.

### 2.4 Gas exchange and photosynthetic rate

The light-saturated photosynthetic rate and gas exchange of completely grown leaves were measured using (LI-COR LI-6400, LI-COR Inc., Lincoln, NE, USA) as described by AbdElgawad et al. (2015). Noncyclic electron transport photochemical efficiency (Fv/Fm) in PSII was measured. A fluorimeter was used to measure the fluorescence of dark-adapted leaves (leaf number 5 or 6) after 30 minutes (PAM2000, Walz, Effeltrich, Germany). At 14 and 21 DAS, stomatal conductance (gs) was measured *in situ* on leaves (L1,2, and L3,4) using a Leaf Porometer (Model SC-1, Decagon Devices, Inc., Hopkins, Pullman, WA USA). To determine the concentration of the pigments, they were extracted in 80% acetone (AbdElgawad et al., 2015).

### 2.5 Evaluating the pigment content

The pigment contents in wheat were isolated and measured by Al Jaouni et al. (2018). Frozen plant materials were extracted in acetone using a MagNALyser (Roche, Vilvoorde, Belgium). After centrifugation at 14,000 g for 20 minutes at 4°C, the supernatant was filtered (Acrodisc GHP filter, 0.45 m 13 mm). HPLC was used to measure the concentrations (Shimadzu SIL10-ADvp, reversed phase). A diode array detector (Shimadzu SPD- M10Avp) was used to measure chlorophyll a, chlorophyll b, and Carotenoids at 420, 440, and 462 nm.

### 2.6 Oxidative stress indicators

To assess lipid peroxidation levels, a MagNALyser was used to extract 50 mg tissues (leaves and well-cleaned root segments) in 1 mL 80% ethanol (Roche, Vilvoorde, Belgium). The thiobarbituric acid test was then used to determine the extract’s malondialdehyde (MDA) level (Hodges et al., 1999). Protein Carbonyl Colorimetric Assay Kit from Cayman Chemical (Ann Arbor, MI) was utilized to measure protein carbonyls as oxidative damage markers (Levine et al., 1994). The Xylenol orange approach was used to quantify hydrogen peroxide (H_2_O_2_) in a trichloroacetic acid (TCA) (0.1%) extract of plant materials (Jiang et al., 1990). Each sample was compared to its catalase (CAT) treated counterpart to reduce non-specific interactions.

### 2.7 Antioxidant metabolite identification

TAC (ferric reducing antioxidant power, FRAP) was measured in ice-cold 80% ethanol with a MagNALyser (Roche, Vilvoorde, Belgium) and quantified using Trolox as a reference, as reported by Benzie and Strain (1999). Plant samples were extracted in 80% ethanol and measured for ascorbate and glutathione using a MagNALyser (Roche, Vilvoorde, Belgium). Reduced ascorbate (ASC) and glutathione (GSH) were measured using HPLC. To extract phenols and flavonoids, fresh plant materials were homogenized in 80% ethanol before being centrifuged at 5000 rpm for 15 minutes. The clear extract was then used to measure the phenols and flavonoid concentrations using the Folin-Ciocalteu and aluminum chloride assays, respectively (AbdElgawad et al., 2023). Tocopherols were extracted with hexane (100 mg FW in 6 ml hexane) and centrifuged for 15 minutes at 14,000 g. The extracts were dried (Labconco, Kansas, USA) and resuspended in hexane. HPLC (Shimadzu, Hertogenbosch, the Netherlands) was used to separate and quantify tocopherols under normal phase conditions. Particil Pac 5 mm column material, length 250 mm, i.d. 4.6 mm, and internal standard dimethyl tocol (DMT) (5 ppm). The Shimadzu Class VP 6.14 software was used to examine the data.

### 2.8 Evaluation of antioxidant enzyme activities

Wheat plant materials (100 mg) were extracted in 1 ml of buffer [50 mM potassium-phosphate, pH 7.0, 10% (w/v) polyvinyl pyrrolidone (PVP), 0.25% (v/v) Triton X-100, 1 mM phenylme- thylsulfonyl fluoride (PMSF), 1 mM ASC] using a MagNALyser (Roche, Vilvoorde, Belgium). The clear supernatant was used to test the activities of superoxide dismutase (SOD, EC 1.15.1.1), peroxidase (POX, EC 1.11.1), catalase (CAT, EC1.11.1.6), glutathione peroxidase (GPX, EC 1.11.1.9), ascorbate peroxidase (APX, EC 1.11.1.11), glutathione reductase (GR). SOD activity was measured by the inhibition of nitroblue tetrazolium (NBT) reduction at 560 nm, as described by Dhindsa et al. (1982). The oxidation of pyrogallol (430 = 2.47 mM-1.cm-1) was used to measure POX activity (Kumar and Khan, 1982). The breakdown of H_2_O_2_ at 240 nm (240 = 0.0436 mM^-1^.cm^-1^) was used to assess CAT activity (Hugo and Lester, 1984). Murshed et al. (2008) described how to quantify APX, MDHAR, DHAR, and GR activities. Drotar and Fall (1985) defined GPX activity as a reduction in NADPH absorbance at 340 nm (340 = 6.22 mM^-1^.cm^-1^).

Soluble protein was measured using the Lowry et al. (1951) technique.

### 2.9 Statistical analysis

Each study was set up in triplicate using a completely randomized design. The data are represented as (means standard error) using the GraphPad Prism version 8.4.2. The statistical analysis used one-way ANOVA (*post-hoc* TukeyHSD (*p* ≤ 0.05). Each experimental value was compared to the appropriate control value.

## 3 Results and discussion

Here, the effect of ambient CO_2_ on wheat was investigated compared to the negative impact of Sb at high concentrations. We tested the idea that high concentrations of Sb could hurt wheat’s growth and bioactive metabolites, but that eCO_2_ (720 ppm) treatment could make up for this and help wheat grow. First, we investigated the effects of Sb at 1500 mg/kg soil on growth, photosynthetic pigments, antioxidant capacity, and minerals in wheat plants. Sb induces detrimental effects on plant growth reduction and photosynthetic activity and increases the formation of ROS, and lipid peroxidation, at moderate and high concentrations (Vidya et al., 2022). Due to inadequate research, high concentrations effects of Sb on wheat will also have an impact on the crop’s yield and farmer’s income. Consequently, it is crucial to investigate the impact and phytotoxicity of Sb treatment and how this effect will be altered under future climate CO_2_ conditions.

### 3.1 Elevated CO_2_ mitigates growth reduction by reducing Sb uptake and improving photosynthesis activity and mineral contents

Plant growth is known to be significantly impacted by Sb accumulation (Zhou et al., 2018). Similarly, the phytotoxicity effect of Sb at 1500 mg/kg soil heavy metal on wheat growth was recorded (**Table 1**). Whereas the fresh weight (FW) and dry weight (DW) of wheat were approximately decreased by Sb treatment (2.4 and 2.15-fold, respectively). In line with our findings, the growth of *Acorus calamus* was substantially hindered by raising the Sb concentration to 1000 and 2000 mg/kg in soil (Zhou et al., 2018). In addition, the cytotoxic effect of Sb at concentrations above 10 mg/L on the seedlings of *Brassica napus* L. and *Raphanus sativus* L. radish significantly decreased the germination rate and germination index (Liang et al., 2018). The effects of aCO_2_ and eCO_2_ were approximately close and there are no significant differences between both treatments. On the other hand, eCO_2_ treatment significantly increased FW and DW of wheat plants. Moreover, we examined whether eCO_2_ treatment could mitigate the detrimental effects of Sb on wheat growth (FW and DW) and physiology (**Table 1**). The stress mitigation impact of eCO_2_ has been previously reported (Ahiahonu et al., 2022; Wang et al., 2023). Interestingly, eCO_2_ has been suggested as an effective approach to mitigate heavy metals stress induced growth inhibition and oxidative stress (Dong et al., 2020; AbdElgawad et al., 2023; Wang et al., 2023). Similarly, the eCO_2_- Sb co-application significantly alleviated the negative impact of Sb on wheat growth (*p* < 0.05). eCO_2_ ameliorated the negative impact of Sb stress and increased the fresh and dry weights by 1.68 and 1.6-fold, respectively compared to Sb treatment. In line with our results, Fernandez et al. (2018) showed that eCO_2_ treatment lowered As concentration in *Arabidopsis thaliana* tissues, resulting in a significant increase in total biomass. Moreover, eCO_2_ significantly increased biomass accumulation by 1.46, 1.47, and 2-fold in the three Alfalfa Haraz, Khider, and Rajab cultivars, respectively (Alotaibi et al., 2021).

**Table 1:** Effect of eCO_2_ either alone or in combination with antimony (Sb) upon the uptake of antimony as well as the biomass (FW and DW) of wheat plants grown in soils contaminated with antinomy.

	aCO_2_	Sb	eCO_2_	eCO_2_+Sb
**Antimony uptake (µg/gDW)**	0.0 ± 0.00^c^	252.6 ± 3.59^a^	0.0 ± 0.00^c^	148.0 ± 2.37^b^
**Fresh weight (g/plant)**	9.03 ± 0.22^a^	3.74 ± 0.12^c^	9.11 ± 0.19^a^	6.32 ± 0.22^b^
**Dry weight (g/plant)**	0.99 ± 0.03^a^	0.46 ± 0.01^c^	1.08 ± 0.02^a^	0.74 ± 0.03^b^

The values in the table represent the mean of at least 3 replicates ± standard errors. Different letters indicate statistically significant difference between the means assessed by *post-hoc* TukeyHSD (*p* ≤ 0.05).

**Figure 1:**
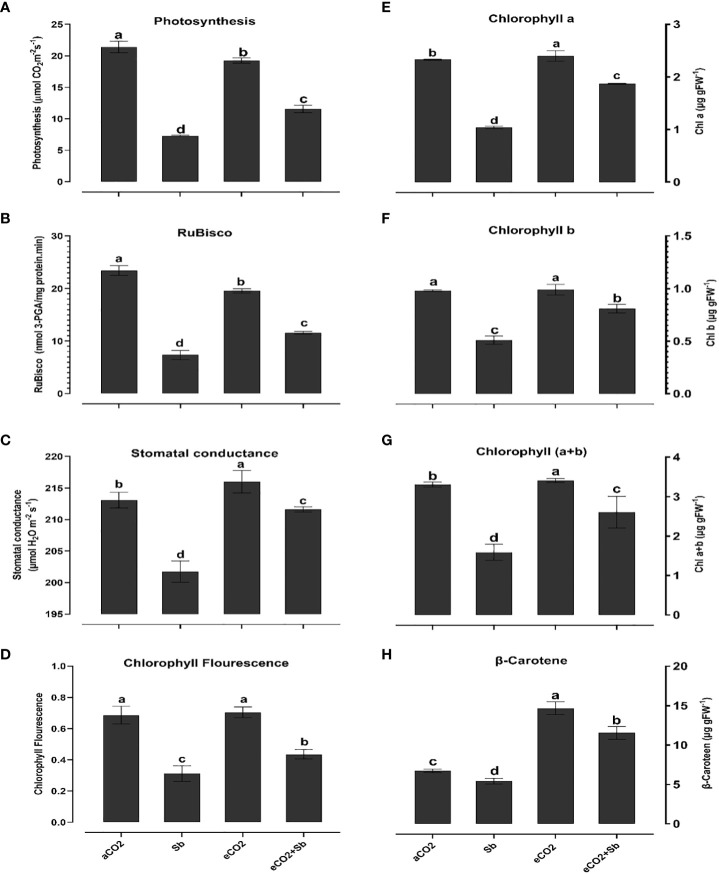
Effect of eCO_2_ either alone or in combination with antimony (Sb) upon the photosynthetic efficiency of wheat plants grown in soils contaminated with antimony, **(A)** photosynthesis, **(B)** Chlorophyll a, **(C)** Chlorophyll b, **(D)** Chlorophyll (a+b), **(E)** RuBisco, **(F)** β-Carotene **(G)** Gas exchange, **(H)** Chlorophyll fluorescence. The values in the table represent the mean of at least 3 replicates ± standard errors. Different letters indicate statistically significant difference between the means assessed by *post-hoc* TukeyHSD (*p* ≤ 0.05).

High CO_2_ plays a beneficial role in mitigating heavy metal toxicity, primarily by stimulating phototypesets, especially within C_3_ plant systems (Fernandez et al., 2018; AbdElgawad et al., 2020). Thus, to understand the basis of improved growth by eCO_2_ under Sb treatment, we measured the photosynthesis rate, photosynthetic pigments, reactions and efficiency. It is well known that heavy metals negatively affect photosynthesis and chlorophylls synthesis and structural degradation (Zhang et al., 2011). Our findings showed that wheat plants under Sb stress revealed a decline in photosynthesis rate, Rubisco activity and levels of chlorophyll and carotene, and an increase in stomatal conductance and chlorophyll florescence (**Figures 1A–H**). The decreases in photosynthesis rate, Cha, Chb and Cha+B levels, and Rubisco activity were 35%, 44%, 51%, 47% and 45%, compared to the corresponding aCO2, respectively (**Figures 1A, B, E, F, G**). In line with our results, Zhou et al. (2018) revealed that high-concentration Sb stress (500-2000 mg/kg) reduced net photosynthetic rate (Pn), leaf pigment (carotenoid and Cha, and Chb), stomatal conductance (gs) and PSII electron transfer quantum yield rate which significantly contributed to the observed decline in *A. calamus* growth. In this regard, Luo et al. (2021) reported that high Sb levels in plant cells can react with the sulfhydryl group of chloroplast proteins, disrupting the structures and functions of chloroplasts, reducing photosynthetic efficiency, and finally decreasing plant growth. In addition, under Sb stress, gas exchange, and chlorophyll fluorescence (Fv/Fm) (**Figures 1C, D**) were all significantly reduced which represented 93, and 58% compared to the corresponding aCO_2_ treatments, respectively. The Fv/Fm ratio decreased as the Sb concentration increased to 2000 mg, suggesting that the PSII reaction centers were destroyed (Zhou et al. (2018). At gas exchange level, Sb treatments gas reduced gs which can account for the decline in photosynthesis under stress conditions. As a consequence, the rate of photosynthesis is hampered, and the overall growth and productivity of the plant, such as in the case of wheat plants, are adversely affected (AbdElgawad et al., 2023).

In contrast, in response to eCO_2_ alone, as compared to Sb stress, the rate of photosynthetic activity and Cha, and Chb were significantly increased (p < 0.05). eCO_2_-Sb co-application also mitigated the reduction in Rubisco and the increase in chlorophyll fluorescence was significantly increased by 1.4 and 1.37-fold compared to the corresponding Sb treatments, respectively (**Figures 1B, D**). High CO_2_ levels are known to increase photosynthesis, which sustains carbon skeletons and provides the energy necessary for plant growth and development (AbdElgawad et al., 2020). High CO_2_ can also reduce the inhibitory effect of abiotic stressors on the rate of carboxylation by Rubisco and the regeneration capacity of RuBP (Guo et al., 2015). Improved Rubisco by eCO_2_ enhances C fixation and, hence, increase the supply of the energy required for plant growth (Al Jaouni et al., 2018; AbdElgawad et al., 2019). Interestingly, Guo et al. (2015) revealed that eCO_2_ boosted plant growth by boosting leaf photosynthetic pigments, particularly at high levels of Cd exposure. Except for all pigments, β carotenoids contents were significantly increased under Sb stress and represented 2.7 and 1.26-fold the corresponding eCO_2_ and eCO_2_-Sb co-application, respectively (**Figure 1H**). Increasing the content of β carotenoids under Sb treatment and decreasing under eCO_2_ treatment explained that wheat plants produce high content of β carotenoids to minimize Sb induced oxidative damages. β-carotene potential for scavenging the dangerous reactive oxygen species in addition to their critical roles in the photosynthetic process is well known (Young, 1991).

High CO_2_ is known to affect stomatal conductance under control and stress conditions (AbdElgawad et al., 2021). The decrease in stomatal conductance may slow down the transpiration, which decreases heavy metals absorption and transport to barley shoots under eCO_2_, especially. The transfer of nutrients and metals from roots to shoots heavily depends on transpiration and root pressure (Mirza et al., 2014). Sb accumulates mostly in roots, which may interfere with plant cation absorption and chlorophyll production, as well as inhibit the transfer of certain nutritional components into leaves. To address the connection between the decreases in shoot DW, FW, and the accumulation of Sb in wheat tissues. The levels of Sb heavy metal in wheat shoots under both Sb-1500 mg/kg soil and eCO_2_-Sb-1500 mg/kg soil were assessed. Our results revealed that Sb was more accumulated in Sb-treated wheat, but it was significantly reduced by eCO_2_-Sb co-application (**Table 1**). The mineral contents were evaluated in the current study to get a better understanding of the link between mineral contents and Sb accumulation. Herein, the minerals’ contents under Sb treatment significantly decreased (p<0.05) and the highest minerals levels were recorded in eCO_2_-treated wheat followed by the mineral’s contents under aCO_2_ conditions (**Table 2**). The reduction in mineral contents such as P, S, K, Mg, Ca, and Zn under Sb stress represented 61, 68, 72, 65, 65, and 46% of the corresponding aCO_2_ treatments, respectively (**Table 2**). Consequently, in wheat seeds, an increase in Sb induced a reduction in K and Mg levels (Shtangeeva et al., 2012). eCO_2_-Sb-co-application diminished the Sb harmful impact by a significant increase (p<0.05) in the mineral’s contents. Hereby, the levels of P, S, K, Mg, Ca, and Zn represented 1.5, 1.41, 1.38, 1.65, 1.66, and 1.5-fold compared to the corresponding Sb treatments, respectively (**Table 2**). In line with our findings, eCO_2_ treatment increased the absorption of micronutrients and macronutrients (Noguchi et al., 2015). Increased CO_2_ is also affected soil chemical and biological conditions. Increased acidic root exudates like citric and malic acids decreased heavy metals bioavailability to roots (AbdElgawad et al., 2023). For example, organic acids acting as a chelating agent, malate binds to toxic metal ions in the rhizosphere or apoplast, effectively preventing their entry into the cytoplasm (Nigam et al., 2003). Increased mineral absorption by plants, particularly under eCO_2_ levels, can contribute to mitigating Sb harmful effects. In this context, eCO_2_ increased root exudates into the soil, which in turn improves soil structure and nutrient retention. This also decreased the bioavailability heavy metals (Guo et al., 2012). These changes can indirectly aid in reducing Sb bioavailability and mineral uptake (Shtangeeva et al., 2012). To summarize, the observed increase in biomass accumulation in wheat plants treated with eCO_2_ can be attributed to the reduction in Sb accumulation, which in turn alleviates the inhibition of photosynthesis. Consistently, the rate of photosynthetic rate, activity, as well as the levels of Cha, Chb, photosystem II efficiency and Rebuisco enzymes activity, exhibited a significant increase under eCO2+SB conditions compared to the conditions with Sb stress.

**Table 2:** Effect of eCO_2_ either alone or in combination with antimony (Sb) upon the mineral content of wheat plants grown in soil contaminated with antimony.

µg/gFW	aCO_2_	Sb	eCO_2_	eCO_2_+Sb
**Phosphorus (P)**	92.11 ± 3.01^ab^	56.61 ± 1.41^c^	115.4 ± 1.6^a^	87.0 ± 2.55^b^
**Sulphur (S)**	44.91 ± 1.01^b^	30.74 ± 0.34^c^	57.7 ± 1.37^a^	43.57 ± 0.96^b^
**Potassium (K)**	744.2 ± 16.1^b^	540.4 ± 11.1^c^	1037 ± 15.2^a^	750 ± 12.1b
**Magnesium (Mg)**	186.2 ± 5.12^b^	122.3 ± 2.21^c^	255.2 ± 2.58^a^	202.5 ± 8.12^b^
**Calcium (Ca)**	9.591 ± 0.23^b^	6.31 ± 0.08^c^	15.68 ± 0.53^a^	10.48 ± 0.33^b^
**Sodium (Na)**	16.91 ± 0.31^a^	17.02 ± 0.28^a^	17.29 ± 0.37^a^	16.17 ± 0.41^a^
**Zink (Zn)**	6.581 ± 0.07^a^	3.06 ± 0.05^c^	6.12 ± 0.08^a^	4.62 ± 0.11^b^

The values in the table represent the mean of at least 3 replicates ± standard errors. Different letters indicate statistically significant difference between the means assessed by *post-hoc* TukeyHSD (*p* ≤ 0.05).

### 3.2 Elevated CO_2_ improved the redox status in Sb stressed wheat plants

**Figure 2:**
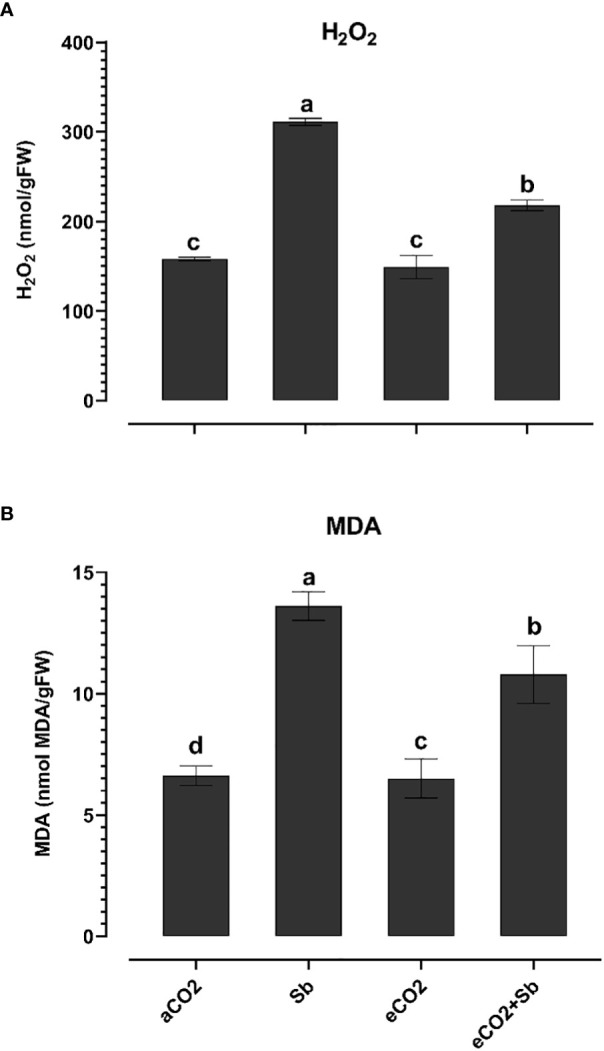
Effect of eCO_2_ either alone or in combination with antimony (Sb) upon the oxidative damage, **(A)** H_2_O_2_ and **(B)** MDA of wheat plants grown in soils contaminated with Sb. The values in the table represent the mean of at least 3 replicates ± standard errors. Different letters indicate statistically significant difference between the means assessed by *post-hoc* TukeyHSD (*p* ≤ 0.05).

**Figure 3:**
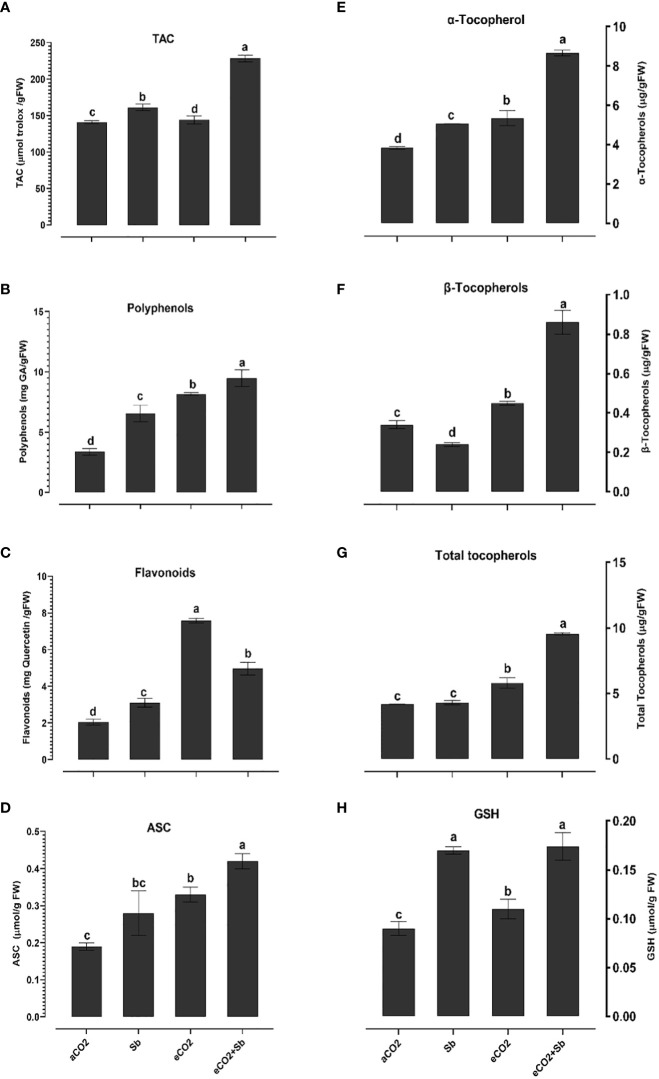
Effect of eCO_2_ either alone or in combination with antimony (Sb) upon the levels of molecular antioxidants, **(A)** total antioxidant capacity (TAC), **(B)** polyphenols, **(C)** flavonoids, **(D)** ascorbate (ASC), **(E)** α- tocopherol, **(F)** β-tocopherol, **(G)** total tocopherols, **(H)** glutathione (GSH) in wheat plants grown in soils contaminated with antimony. The values in the table represent the mean of at least 3 replicates ± standard errors. Different letters indicate statistically significant difference between the means assessed by *post-hoc* TukeyHSD (*p* ≤ 0.05).

**Figure 4:**
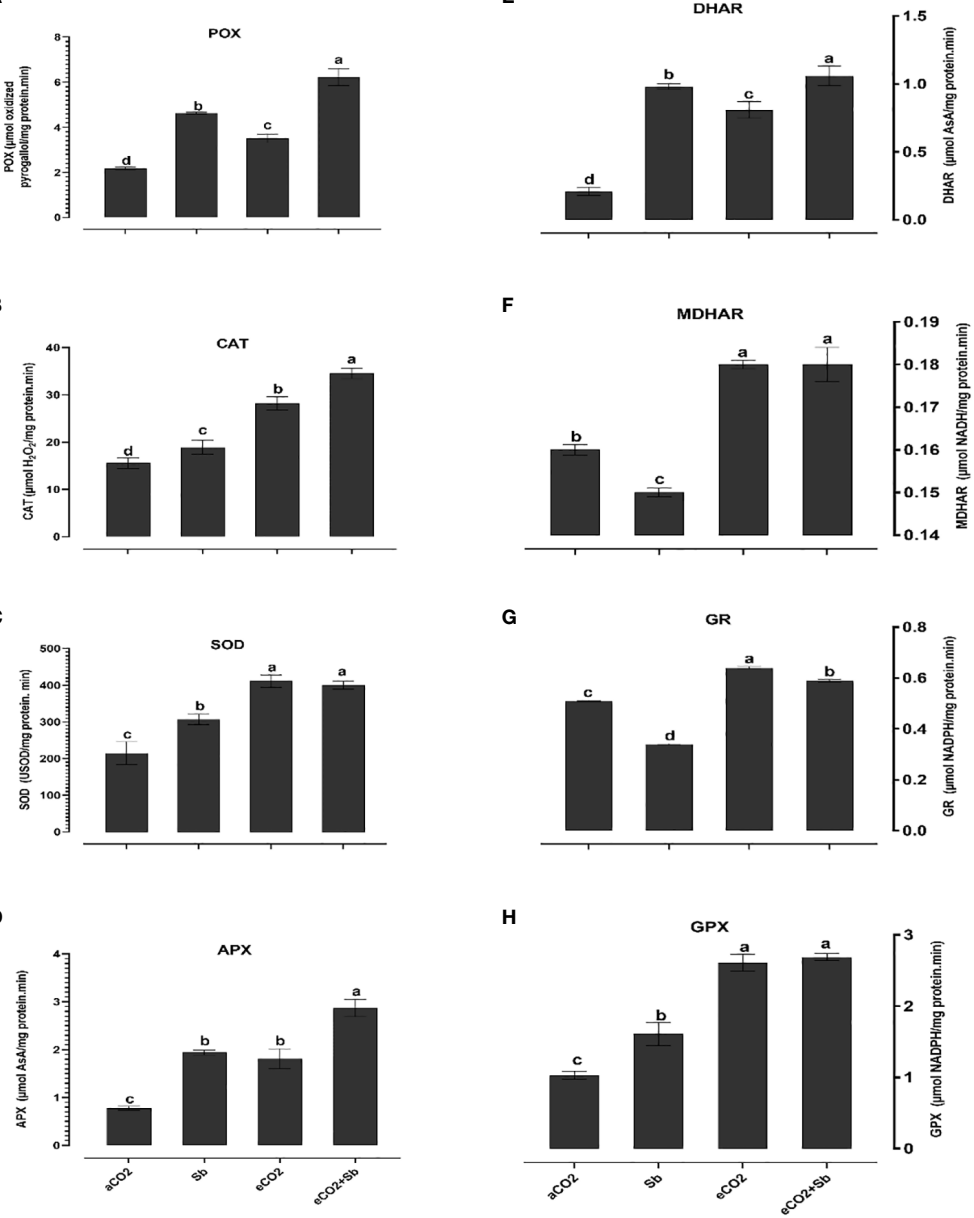
Effect of eCO_2_ either alone or in combination with antimony (Sb) upon the levels of enzymatic antioxidants, **(A)** peroxidase (POX), **(B)** catalase (CAT), **(C)** superoxide dismutase (SOD), **(D)** ascorbate peroxidase (APX), **(E)** dehydroascorbate reductase (DHAR), **(F)** monodehydroascorbate reductase (MDHAR), **(G)** glutathione reductase, **(H)** glutathione peroxidase (GPX) in wheat plants grown in soils contaminated with antimony. The values in the table represent the mean of at least 3 replicates ± standard errors. Different letters indicate statistically significant difference between the means assessed by *post-hoc* TukeyHSD (*p* ≤ 0.05).

**Figure 5:**
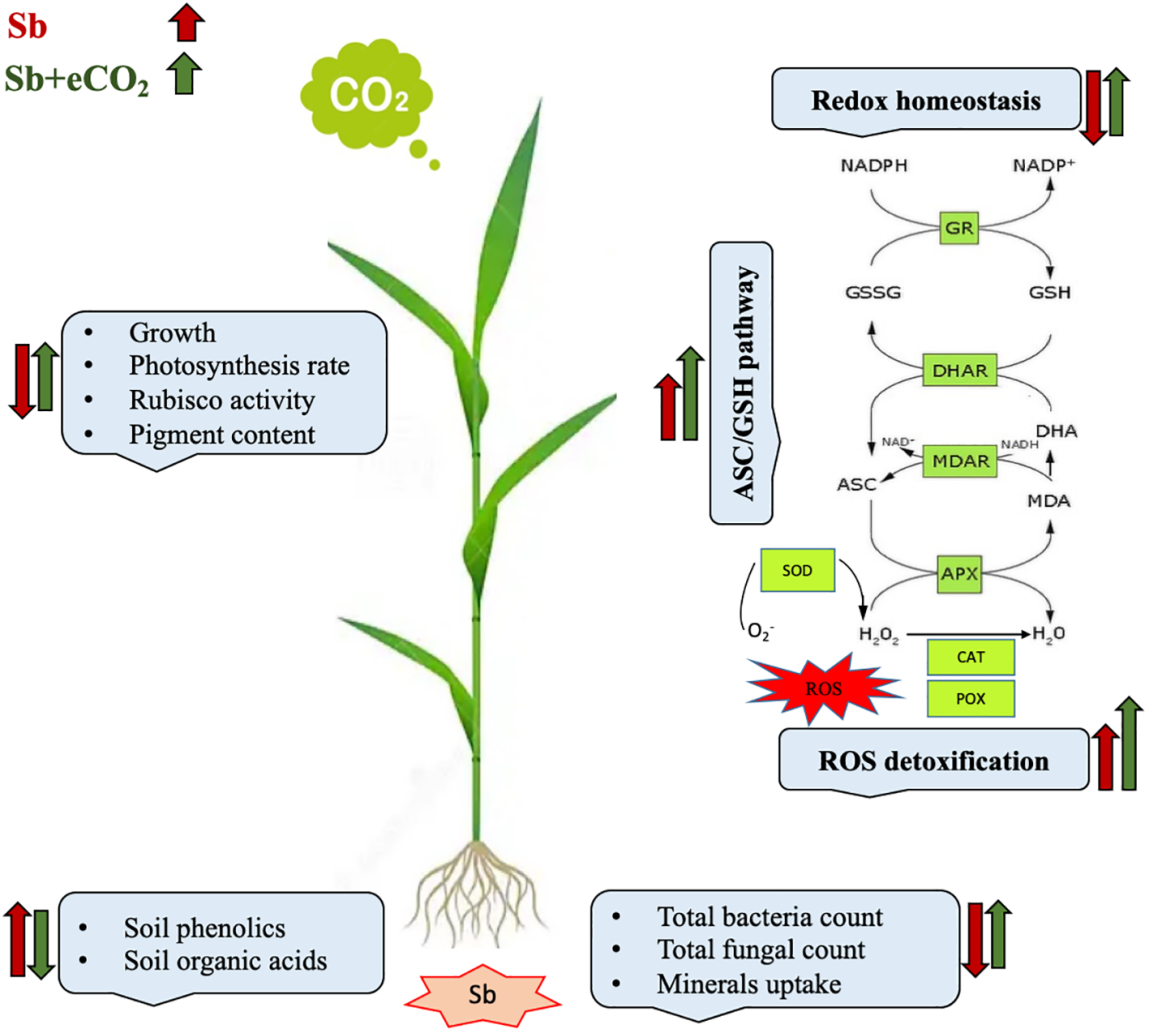
A model explaining the growth, physiological and biochemical responses of wheat plant to antimony (Sb) stress under current and future climate CO_2_ (eCO_2_). Increases or decreases in growth, soil organic acid, photosynthesis, mineral uptake, antioxidant metabolites and enzymes are indicated by a red (Sb effect) or a green (Sb+eCO_2_ effect) arrow. Ascorbate (ASC), glutathione (GSH), peroxidase (POX), catalase (CAT), superoxide dismutase (SOD), ascorbate peroxidase (APX), dehydroascorbate reductase (DHAR), monodehydroascorbate reductase (MDHAR), glutathione reductase, glutathione peroxidase (GPX).

It is well known that high concentrations of heavy metals such as Sb can disrupt the functioning of mitochondria and chloroplasts and disturb the redox equilibrium. As a consequence, this disturbance leads to ROS accumulation, which induces oxidative stress (Cuypers et al., 2016). Similary, Sb treatment significantly induced oxidative damages (p<0.05) as indicated by increased levels of H_2_O_2_ and malondialdehyde (MDA, a marker of ROS-induced lipid peroxidation) (**Figures 2A, B**). According to Luo et al. (2021), there were significant elevated levels of H_2_O_2_ and MDA in Sb-treated rice seedlings, which resulted in growth suppression and disruption of redox homeostasis. Over accumulation of Sb in plants may explain the oxidative stress in wheat plants. According to Cuypers et al. (2016), under conditions of heavy metal stress, a plant’s priming, and storage of H_2_O_2_ in its roots may serve as a defense mechanism. Under stress conditions, plants generate a series of antioxidant enzymatic defense mechanisms to remove ROS and protect plants from oxidative damage (Guo et al., 2015; Khamis et al., 2019). To understand the basis of Sb stress mitigation effect of eCO_2_, we investigated their effect on the content of antioxidant metabolites. The exposure to eCO_2_-Sb co-application caused a significant decrease in H_2_O_2_, and MDA oxidative stress markers by 1.96 and 2.8, respectively. In line with our results, severe As treatment under aCO_2_ induced protein oxidation. This showed that the tissues’ antioxidant capacity was increased by the eCO_2_ exposure (AbdElgawad et al., 2021). During environmental stress, it has been demonstrated that eCO_2_ reduces the accumulation of MDA in *Lolium* species (Farfan-Vignolo and Asard, 2012). Under eCO_2_, plants employ organ-specific antioxidant arsenal components to resist oxidative stress (AbdElgawad et al., 2020). Herein, Sb treatments and eCO_2_-Sb-co-application dramatically boosted ROS scavenging enzyme activities in response to Sb toxicity in wheat plants compared to eCO_2_ and aCO_2_ conditions. The current findings showed that levels of antioxidant metabolites such as TAC, polyphenols, flavonoids, GSH ASC (**Figures 3A–D, H**) and antioxidant enzymes such as POX, CAT, SOD, APX, DHAR, MDHAR, GR, and GPX (**Figures 4A–H**) signifcantly increased the Sb effect compared to the aCO_2_. eCO_2_-Sb-treated wheat had significantly recorded (p<0.05), the highest levels in comparison to other treatments. Consequently, the effect of oxidative stress has been observed to be reduced in many plant species by increasing the activity of antioxidant enzymes (Cuypers et al., 2016). Moreover, POX, CAT, and SOD levels in eCO_2_-Sb-treated wheat were significantly increased by 2.87, 2.2, and 1.86-fold, respectively, compared to the corresponding aCO_2_ treatment (**Figures 4A–C**). In line with what we found, the CAT, APX, MDHAR, and GR activities of barley roots increased significantly when they were treated with eCO2 and put under As stress compared to when they were treated with aCO2 and put under As stress (AbdElgawad et al., 2021). Barley’s photorespiration, H_2_O_2_ generation, and lipid/protein oxidation were all decreased by exposure to eCO_2_. These results imply that both Sb-treated wheat and eCO_2_-Sb-co-application treatment under study were able to maintain a balanced antioxidant system for detoxification of excess ROS caused by Sb. The ascorbate-glutathione (ASC/GSH)-mediated antioxidative defense mechanism was upregulated in maize because of exposure to eCO_2_ (AbdElgawad et al., 2021). Where eCO_2_ treated wheat significantly recorded (p<0.05) the highest SOD activity (**Figure 4C**). To combat the damaging effects of ROS, plants use antioxidant defense mechanisms. SOD serves as the first line of defense against oxidation (Luo et al., 2021). In our study, wheat plants that had been exposed to Sb and eCO_2_-Sb-co-application treatments had the highest induction of CAT, POX, and SOD compared to eCO_2_ and aCO_2_ conditions (**Figure 5**). Also, it was suggested that increasing SOD levels in maize tissues exposed to heavy metals was a protective mechanism and that increased GR activity indicates the regeneration of reduced glutathione, which may have been directly damaged by ROS (AbdElgawad et al., 2020). Furthermore, Jia et al. (2010) showed that eCO_2_ greatly boosted SOD, APX, and GR activities in J172 leaves grown in high Cd-contaminated soil. This finding implies that eCO_2_ can reduce oxidative damage by enhancing antioxidant enzyme activity in J172 leaves (Guo et al., 2015). Moreover, relative to the corresponding aCO_2_, alpha, beta and total tocopherol levels in eCO_2_-Sb-co-application significantly increased (p<0.05) by 2.25, 2.6 and 2.28-fold, respectively. Treated wheat plants under Sb stress and eCO_2_-Sb-co-application treatments displayed the highest amounts of both phenols and flavonoids in comparison to aCO_2_ and eCO_2_ treatments, and the levels of phenols and flavonoids were significantly (p<0.05) higher under Sb treatment. In summary, studying the interaction between eCO_2_ and Sb on antioxidant responses is important to understand their role in environmental stress mitigation (**Figure 5**).

### 3.3 Elevated CO_2_ improved soil chemical composition and health

The evaluation of the impact of CO_2_ and Sb on organic acid levels and microorganisms in the soil is a crucial study that can provide insights into how these factors influence soil health and microbial communities. Here, eCO_2_-Sb-co-application released the Sb from the wheat plant under treatment and significantly increased (p<0.05) the Sb levels on the soil by 1.43-fold compared to the corresponding Sb treatments (**Table 3**). Plants have developed several defenses against the harmful effects of toxic metals, including the exudation of complexing agents to reduce metal uptake and alter the kinetic characteristics of transporters. To maintain metal-ion homeostasis, plants control metal influxes and effluxes by considering the needs of the entire plant (Shtangeeva et al., 2012). Sb significantly decreased the levels of soil contents of phenols, total bacteria, and fungi. The highest soil content of phenols and total bacteria and fungi were recorded under eCO_2_-Sb-co-application followed by eCO_2_ treatments. The contents of phenol, total bacteria, and fungi in soil under eCO_2_ and Sb co-application were significantly increased by 1.38, 1.25, and 1.42-fold, respectively. In line with our results, AbdElgawad et al. (2021) reported that application of eCO_2_ minimize As^III^ accumulation by increasing the polyphenol and citric acid exudation into the soil. The ability of eCO_2_ to alter the chemical composition of the rhizosphere was reported by Fernandez et al. (2018). In this context, eCO_2_ treatment increased organic acid exudation by roots, and these exudates can act as electron transporters or as ligands for metal binding to decrease heavy metal’s availability. Adversely, Sb-stress significantly increased the soil contents of citric and oxalic acid by 1.29 and 1.15-fold compared to the eCO_2_-Sb-co-application and 1.2 and 1.73-fold compared to their corresponding aCO_2_ treatment, respectively. In line with our findings, AbdElgawad et al. (2021) reported that the citrate and phenolic content in the soil were increased under As and eCO_2_ conditions compared to the corresponding levels under aCO_2_ may be, at least in part, responsible for the reduction in heavy metal accumulation in eCO_2_-treated plants. Similar findings by Phillips et al. (2009) and Sanchez-Carrillo et al. (2018) demonstrated that eCO_2_ treatment increases root exudates of organic acid from plants, perhaps through altering carbon allocation. In addition, organic compounds like phenols and organic acids can serve as ligands for binding metals and as electron carriers transferring electrons with Sb (Campbell and Nordstrom, 2014). The role of soil microorganisms in heavy metal mitigation was reported according to Huang et al. (2020), who showed that eCO_2_-enriched soil microbiota counteracted the effects of Cd on *Robinia pseudoacacia*, concerning further eCO_2_ accumulation in shoots. Furthermore, microorganisms can reduce their intake of hazardous substances to survive in the presence of undesirable substances. In addition, it may be assumed that Sb was not strongly linked to the organic molecules of the plant cells following such a quick uptake of Sb by the plants.

**Table 3:** The changes in the levels of antinomy, phenols, citric acids, oxalic acids, total bacteria, and total fungi in the soils of wheat plants grown in soils contaminated with Sb under ambient and elevated levels of CO_2_.

mg/gFW	aCO_2_	Sb	eCO_2_	eCO_2_+Sb
**Antimony/Soil**	00.00 ± 0.0^c^	846.8 ± 24.1^b^	00.00 ± 0.0^c^	1216 ± 27.1^a^
**Phenol/Soil**	92.88 ± 3.2^c^	101.1 ± 1.82^b^	109.9 ± 6.13^a^	140.9 ± 3.81^a^
**Citric acids**	246.2 ± 5.12^c^	384.64 ± 3.8^a^	236.3 ± 1.61^c^	296.7 ± 4.41^b^
**Oxalic acid**	92.81 ± 3.34^d^	161.1 ± 1.81^a^	119.9 ± 6.51^c^	140.9 ± 3.94^b^
**Total bacteria**	146.46 ± 5.15^d^	184.1 ± 18.13^c^	222 ± 6.14^a^	231.4 ± 4.14^b^
**Total Fungi**	101.12 ± 7.34^d^	131.2 ± 8.45^c^	159 ± 5.14^b^	187.5 ± 17.4^a^

The values in the table represent the mean of at least 3 replicates ± standard errors. Different letters indicate statistically significant difference between the means assessed by *post-hoc* TukeyHSD (*p* ≤ 0.05).

In summary, when subjected to Sb stress, the elevated levels of phenol, citric acid, and oxalic acid can enhance the availability of Sb in the soil. However, under elevated CO_2_ conditions, the increased levels of microorganisms in the soil may counteract this effect by mitigating Sb availability (**Figure 5**). As a result, the harmful impact of Sb on the treated wheat plant is diminished.

## 4 Conclusion

In the current study, antimony at 1500 mg/kg, soil showed a harmful effect on the wheat plant through a significant reduction in the growth and photosynthesis rate combined with an increase in the levels of carotenoids and enzymatic antioxidants (**Figure 5**). Adversely, eCO_2_ significantly increased the growth and photosynthesis pigments such as Chl a and Chl b and induced the enzymatic and non-enzymatic antioxidants and minerals contents of treated wheat. Using the eCO_2_-Sb-co application was reported to mitigate the harmful effect of Sb by restoring the increase in growth parameters and photosynthesis rate. Furthermore, increasing the levels of antioxidants to counteract the presence of ROS and increasing the mineral contents and soil substances minimized the availability of Sb in the soil and reduced the harmful effect of Sb on the wheat plant under investigation (**Figure 5**).

## Data availability statement

The original contributions presented in the study are included in the article/supplementary material. Further inquiries can be directed to the corresponding author.

## Author contributions

GK: formal analysis, conceptualization, writing—original draft; AM: writing—review and editing, data curation; EA: formal analysis, data curation; SK: data curation, HA: data curation; conceptualization, review, and editing, HAE: writing, formal analysis, data curation; conceptualization, review, and editing.
